# Effect of *compound kushen injection* on immune function in patients with colorectal cancer: a systematic review and meta-analysis

**DOI:** 10.3389/fphar.2025.1565031

**Published:** 2025-04-03

**Authors:** Lixin Zhang, Guangyan Wei, Kaiping Wang, Xu Han

**Affiliations:** ^1^ Department of Anorectal Surgery, The First Affiliated Hospital of Guizhou University of Traditional Chinese Medicine, Guiyang, China; ^2^ College of Pharmacy, Yanbian University, Yanji, China; ^3^ Department of Anorectal Surgery, Chongqing Changshou Traditional Chinese Medicine Hospital, Chongqing, China

**Keywords:** compound kushen injection, colorectal cancer, immune function, randomized controlled trials, systematic review, meta-analysis

## Abstract

**Background:**

Colorectal cancer (CRC) is one of the most common malignant tumors worldwide. Chemotherapy and radiotherapy remain cornerstone treatments; however, they often lead to significant immune suppression and an increased risk of infection. Enhancing immune function in CRC patients is critical for improving clinical outcomes and prognosis.

**Objective:**

To evaluate the effects of Compound Kushen Injection (CKI) on immune function and its role in mitigating chemotherapy-induced adverse effects in patients with CRC.

**Methods:**

We retrieved randomized controlled trials (RCTs) evaluating the effects of CKI on immune function in patients with CRC from eight Chinese and English databases, up until 31 December 2024. The Cochrane Handbook was used to assess the quality of the included studies. For the meta-analysis, we utilized Review Manager 5.4.1 software. Sensitivity analysis and publication bias assessment were conducted using Stata 17.0 software.

**Result:**

A total of 2,663 patients (1,550 males and 1,113 females) from 30 RCTs were included. Compared to conventional chemotherapy (CC), the combination of CKI with CC significantly enhanced immune function, increasing CD3^+^ levels (MD = 6.15, 95% CI: 4.78 to 7.53, *p* < 0.00001), CD4^+^ levels (MD = 8.05, 95% CI: 6.99 to 9.11, *p* < 0.00001), CD4+/CD8+ levels (MD = 0.36, 95% CI: 0.28 to 0.44, *p* < 0.00001), NK cell levels (MD = 3.60, 95% CI: 2.85 to 4.34, *p* < 0.00001), while reducing CD8^+^ levels (MD = −4.19, 95% CI: −5.11 to −3.27, *p* < 0.00001). CKI also improved the objective response rate (ORR, RR = 1.50, 95% CI: 1.38 to 1.62, *p* < 0.00001) and disease control rate (DCR, RR = 1.15, 95% CI: 1.10 to 1.19, *p* < 0.00001), decreased CEA levels (MD = −1.79, 95% CI: −2.81 to −0.76, *p* = 0.0007) and CA199 levels (MD = −0.73, 95% CI: −1.35 to −0.12, *p* = 0.02), and reduced chemotherapy-induced adverse reactions, including nausea, vomiting, hepatic dysfunction, myelosuppression, neurotoxicity, leukopenia, thrombocytopenia, and mouth ulcers.

**Conclusion:**

Current evidence suggests that the combination of CKI with CC may have beneficial effects on immune function, ORR, DCR, and chemotherapy-induced adverse reactions in CRC patients. However, given the variability in study quality and the absence of disease stage stratification, these findings should be interpreted with caution. Furthermore, the lack of long-term follow-up data limits the understanding of CKI’s impact on survival and quality of life. High-quality, large-scale RCTs with extended follow-up are needed to further assess the long-term efficacy, safety, and clinical applicability of CKI in CRC management.

**Systematic Review Registration:**

https://www.crd.york.ac.uk/PROSPERO/display_record.php?RecordID=632516, identifier CRD42025632516

## 1 Introduction

Colorectal cancer (CRC) is among the most prevalent malignancies worldwide, ranking as the third most common cancer and the second leading cause of cancer-related mortality (Siegel et al., 2023). According to the latest global cancer statistics, CRC accounts for approximately 10% of all new cancer cases, with its incidence steadily increasing in both developed and developing countries (Roshandel et al., 2024). Several risk factors, including unhealthy dietary habits, genetic susceptibility, and sedentary lifestyles, are closely associated with CRC onset (Xi and Xu, 2021). For early-stage CRC, surgical resection remains the primary treatment modality (O'Donnell et al., 2024; Shouki et al., 2025). However, advanced or recurrent disease, adjuvant therapies such as chemotherapy and radiotherapy remain essential. Despite their effectiveness in reducing tumor burden and prolonging survival, these treatments are frequently associated with severe adverse effects, including myelosuppression, gastrointestinal toxicity, and hepatic or renal impairment (Lynch et al., 2024). More notably, chemotherapy and radiotherapy can induce substantial immunosuppression, leading to compromised antitumor immunity, increased susceptibility to infections, and a poorer overall prognosis (Roberti et al., 2020). These challenges underscore the urgent need for adjunctive therapeutic strategies that minimize immunosuppression while effectively countering tumor progression.

Immune function plays a pivotal role in CRC progression (Wan et al., 2024). The immune system exerts tumor-suppressive effects primarily through immunosurveillance mechanisms, with tumor-infiltrating lymphocytes (TILs) and natural killer (NK) cells being key components (Zhang et al., 2024). However, chemotherapy and radiotherapy frequently cause profound immune dysfunction, including TIL depletion and impaired NK cell activity (Sharma et al., 2024; Wang et al., 2019). This immune compromise accelerates tumor progression, enhances invasiveness, and diminishes the body’s ability to control malignancy, thereby exacerbating disease progression, reducing quality of life, and increasing mortality rates (Ding et al., 2024; Nicolini and Ferrari, 2024). During the recovery phase following chemotherapy and radiotherapy, immune suppression further hinders recovery by increasing patients’ susceptibility to infections and complications (Chavez-Dominguez et al., 2021). Consequently, restoring immune function and mitigating treatment-induced immune injuries are critical strategies for improving treatment outcomes and enhancing the quality of life for CRC patients.

Compound Kushen Injection (CKI) is a pharmacopoeia-based botanical drug formulation derived from the roots of *Sophora flavescens* Ait, and it has been used as an adjunctive treatment for cancer in China (Zhang J. B. et al., 2023). CKI primarily contains matrine-type alkaloids, including oxysophocarpine, oxymatrine, sophoridine, and matrine, which are standardized in terms of composition and quality control. It exhibits potent antitumor, anti-inflammatory, and immunomodulatory properties (Dong et al., 2019). Previous studies have demonstrated that CKI inhibits cancer cell proliferation, induces apoptosis, and suppresses angiogenesis through multiple signaling pathways (Sun et al., 2022). Additionally, CKI has been shown to alleviate chemotherapy-induced toxicities and improve quality of life in cancer patients (Gao, 2022). In the context of CRC, CKI is believed to enhance immune function by modulating T lymphocytes, promoting cytokine balance, and improving overall immunocompetence (Li C. et al., 2023). These promising findings necessitate a systematic evaluation of CKI’s effects on immune function in CRC patients to provide robust evidence for its clinical application.

## 2 Methods

### 2.1 Study registration

This systematic review and meta-analysis adhered to the 2020 Preferred Reporting Items for Systematic Reviews and Meta-Analyses (PRISMA) guidelines to ensure methodological transparency and minimize potential biases (Supplementary Material S1) (Page et al., 2021). The study protocol was registered in the PROSPERO database (www.crd.york.ac.uk) under registration number CRD42025632516.

To enhance the accuracy of this study, we referenced the Consensus Statement on Phytochemical Characterization of Plant Extracts (ConPhyMP) for standardized reporting of CKI composition. Furthermore, we followed established guidelines for the scientific nomenclature and standardization of botanical drug constituents. The CKI preparation analyzed in this study was derived from *S. flavescens* Ait. [Fabaceae; Sophorae Flavescentis Radix], with its taxonomic classification verified through the Plants of the World Online (POWO) database (http://www.plantsoftheworldonline.org).

### 2.2 Search strategy

Two independent reviewers (LZ and GW) conducted a comprehensive literature search across eight electronic databases: PubMed, Embase, Cochrane Library, Web of Science, China National Knowledge Infrastructure (CNKI), Wanfang Database (WF), China Science and Technology Journal Database (VIP), and Chinese Biomedical Literature Database (CBM). Additional sources included the Chinese Clinical Trial Registry and reference lists of relevant studies to identify additional eligible studies. The search covered all publications from database inception to 31 December 2024, without language restrictions. The search strategy was developed based on the PICOS framework and included a combination of MeSH terms and free-text keywords such as “Compound Kushen Injection,” “Compound Sophora flavescens Injection,” “colorectal cancer,” “colon cancer,” and “rectal cancer.” Detailed search strategies and results for each database are presented in Supplementary Material S2.

### 2.3 Eligibility criteria

#### 2.3.1 Inclusion criteria

(a) Participants: Patients diagnosed with CRC through pathological examination.
(b) Interventions: Control groups received guideline-recommended conventional chemotherapy (CC) regimens (e.g., FOLFOX4, XELOX) via oral or intravenous administration, while treatment groups received CKI in addition to the control group regimens.
(c) Outcomes: Primary outcomes included immune function markers (CD3^+^, CD4^+^, CD8^+^, CD4+/CD8+, and NK cell). Secondary outcomes included objective response rates (ORR), disease control rate (DCR), carcinoembryonic antigen (CEA), carbohydrate antigen 199 (CA199), and adverse reactions. ORR and DCR were assessed based on WHO criteria for solid tumor response. Tumor response was categorized as complete response (CR, disappearance of all target lesions for ≥4 weeks), partial response (PR, ≥50% tumor reduction for ≥4 weeks), stable disease (SD, <50% reduction without progression for ≥4 weeks), and progressive disease (PD, no reduction or new lesions). ORR comprised CR + PR, while DCR included CR + PR + SD.
(d) Study design: Randomized controlled trials (RCTs).

#### 2.3.2 Exclusion criteria

(a) Non-RCTs, including animal studies, *in vitro* studies, reviews, case reports, or letters.
(b) Studies in which the treatment group included other traditional Chinese medicine formulations in addition to CKI.
(c) Studies lacking data on primary outcomes.
(d) Duplicate publications (only the most comprehensive study with complete data was included).
(e) Studies where the full text was inaccessible online or via email.

### 2.4 Study selection and data extraction

The retrieved studies were managed using EndNote (version 20.6). After removing duplicate records, two reviewers (LZ and GW) independently screened titles and abstracts using predefined criteria to exclude clearly ineligible studies. The full texts of the remaining studies were then reviewed comprehensively to finalize the included studies. Discrepancies were resolved through discussion or by consulting a senior reviewer (XH).

Data extraction was independently performed by two reviewers (LZ and KW) using a standardized data extraction form. The extracted data included:

(1) Basic information: First author, year of publication, study title, and journal.
(2) Baseline characteristics: Sample size, age, gender, disease duration, etc.
(3) Intervention details: Dosage, duration, and frequency of CKI, as well as details of conventional treatment methods.
(4) Outcome measures: All relevant outcomes specified in the studies.

### 2.5 Risk of bias assessment

The methodological quality of included studies was assessed independently by two reviewers (GW and KW) using the Cochrane Risk of Bias Tool 2.0 (Cumpston et al., 2019). The six domains evaluated were: randomization methods, allocation concealment, blinding, incomplete outcome data, selective reporting, and other biases. Each study was classified as having low, unclear, or high risk of bias. Any discrepancies were resolved through consultation with a third reviewer (XH) or mutual discussion.

### 2.6 Statistical analysis

Statistical analyses were conducted using Review Manager (version 5.4.1). Continuous outcomes were expressed as mean differences (MD) with 95% confidence intervals (CIs), while dichotomous outcomes were expressed as risk ratios (RR) with 95% CIs. A fixed-effects model was used for low heterogeneity (*I* ^*2*^ < 50% and *p* > 0.05); otherwise, a random-effects model was applied. Statistical significance was set at *p* < 0.05.

### 2.7 Sensitivity analysis

Sensitivity analysis was performed by sequentially excluding individual studies to evaluate the robustness of the results and identify potential sources of heterogeneity. These analyses were conducted using STATA (version 17.0).

### 2.8 Subgroup analysis

Subgroup analyses were conducted based on intervention duration (<8 weeks or ≥8 weeks) to explore potential sources of heterogeneity. These analyses were also performed using Review Manager (version 5.4.1).

### 2.9 Publication bias

Publication bias was assessed using Egger’s test when sufficient studies (*n* > 10) were included. These analyses were performed using STATA (version 17.0).

## 3 Results

### 3.1 Search results and study characteristics

**FIGURE 1:**
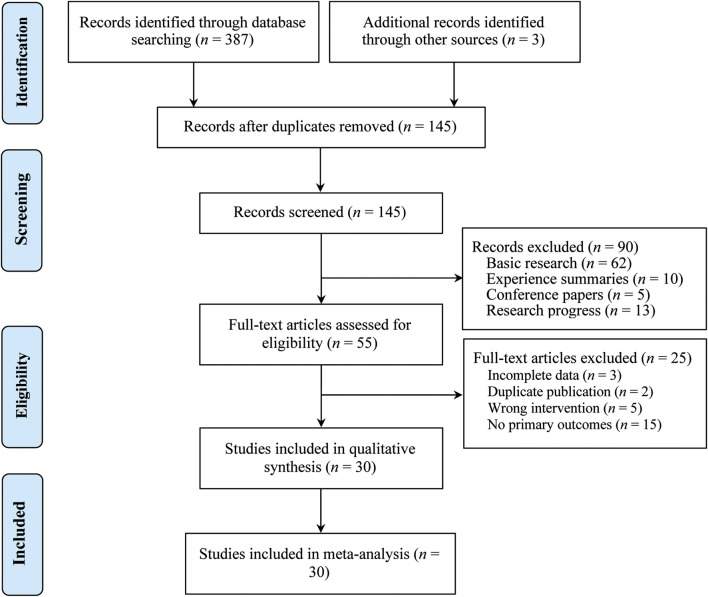
The PRISMA study flowchart of study search.

A total of 390 studies were initially retrieved. After removing duplicates using EndNote software, 90 studies were excluded based on a review of their titles and abstracts. Subsequently, 55 studies underwent full-text review, and 25 studies were excluded. Ultimately, 30 studies (Chen et al., 2009; Ding et al., 2010; He et al., 2023; Kang et al., 2015; Lei, 2022; Li and Ying, 2019; Li, 2022; Li and Yi, 2011; Li, 2021; Liao et al., 2009; Liu and Liu, 2022; Ma et al., 2019; Qiao, 2022; Shi and Zhang, 2023; Song, 2021; Sun et al., 2024; Tong et al., 2018; Wang et al., 2021; Wang Q. J. et al., 2024; Wang, 2020; Xue, 2021; Yin et al., 2020; Yuan, 2016; Zhang et al., 2019; Zhang D. W. et al., 2023; Zhang and Wu, 2017; Zhang and Bai, 2017; Zhao et al., 2022; Zheng et al., 2011; Zhou et al., 2024) met the inclusion criteria and were included in the final analysis (Figure 1). All 30 included studies were conducted in China, involving a total of 2,663 patients (1,550 males and 1,113 females), with publication dates ranging from 2009 to 2024. Sample sizes varied from 20 to 125 participants, and treatment durations ranged from 10 days to 12 weeks. Regarding treatment regimens, 17 studies (Chen et al., 2009; Ding et al., 2010; Kang et al., 2015; Lei, 2022; Li, 2022; Li and Yi, 2011; Liao et al., 2009; Liu and Liu, 2022; Song, 2021; Wang et al., 2021; Wang Z. et al., 2024; Wang, 2020; Yuan, 2016; Zhang et al., 2019; Zhang and Wu, 2017; Zhao et al., 2022; Zheng et al., 2011) utilized CKI in combination with FOLFOX4, seven studies (He et al., 2023; Li and Ying, 2019; Li, 2021; Sun et al., 2024; Tong et al., 2018; Yin et al., 2020; Zhang D. W. et al., 2023) combined CKI with XELOX, one study (Qiao, 2022) used CKI with DP, one study (Shi and Zhang, 2023) applied CKI with RALOX, one study (Ma et al., 2019) combined CKI with Capecitabine, and another one study (Xue, 2021) incorporated CKI with Raltitrexed + Oxaliplatin. Additionally, one study (Zhang and Bai, 2017) involved CKI combined with Oxaliplatin + 5-FU, and one study (Zhou et al., 2024) used CKI in conjunction with Bevacizumab + XELOX. Table 1 provides a detailed summary of the basic characteristics and treatment details of the included studies.

**TABLE 1:** Included studies basic characteristics.

Study ID	Sample size		Sex (M/F)		Mean age (years)		Interventions		Treatment duration	Outcomes
	T	C	T	C	T	C	T	C		
Chen et al. (2009)	40	40	45/35		61.7		C+CKI, 20 mL, qd	FOLFOX4	10d	①②③④⑤
Ding et al. (2010)	30	30	18/12	20/10	64.5		C+CKI, 20 mL, qd	FOLFOX4	12W	①②③④⑤
He et al. (2023)	35	35	20/15	18/17	58.9 ± 16.6	60.9 ± 11.3	C+CKI, 20 mL, qd	XELOX	12W	①②④⑧⑨⑩
Kang et al. (2015)	52	52	55/49		66.31 ± 7.29		C+CKI, 20 mL, qd	FOLFOX4	9W	①②③④⑤⑥⑦
Lei (2022)	77	77	40/37	42/35	53.97 ± 15.22	52.79 ± 14.93	C+CKI, 20 mL, qd	FOLFOX4	12W	①②③④⑥⑦⑩
Li (2021)	40	40	26/14	28/12	53.4 ± 12.3	53.5 ± 12.2	C+CKI, 15 mL, qd	XELOX	10d	②③④⑩
Li (2022)	49	49	27/22	28/21	50.82 ± 4.28	50.60 ± 4.80	C+CKI, 12 mL, qd	FOLFOX4	6W	②③⑥⑦⑧⑨⑩
Li and Yi (2011)	32	36	46/22		55.4		C+CKI, 20 mL, qd	FOLFOX4	8W	①②③④⑤⑥⑦
Li and Ying (2019)	39	39	22/17	25/14	56.8 ± 6.2	57.8 ± 6.4	C+CKI, 12 mL, qd	XELOX	12W	②③④⑤⑥⑦⑧⑨⑩
Liao et al. (2009)	125	125	69/56	73/52	58.6	56.7	C+CKI, 20 mL, qd	FOLFOX4	2W	①②③④⑤⑥⑦
Liu and Liu (2022)	39	39	21/18	26/13	59.13 ± 4.18	58.25 ± 5.67	C+CKI, 20 mL, qd	FOLFOX4	8W	①②③④⑤⑥⑦⑩
Ma et al. (2019)	30	30	21/9	22/8	62.5		C+CKI, 20 mL, qd	Capecitabine	4W	①②③④⑤⑥⑦⑩
Qiao (2022)	59	59	35/24	32/27	62.74 ± 4.32	63.67 ± 4.62	C+CKI, 20 mL, qd	DP	9W	①②③④⑩
Shi and Zhang (2023)	39	39	18/21	21/18	59.03 ± 13.36	56.66 ± 12.85	C+CKI, 12 mL, qd	RALOX	3W	④⑤⑥⑦⑧⑨⑩
Song (2021)	40	40	23/17	24/16	52.49 ± 5.44	50.87 ± 5.17	C+CKI, 15 mL, qd	FOLFOX4	8W	②③④⑥⑦⑩
Sun et al. (2024)	44	40	26/18	28/12	65.0 ± 8.8	63.1 ± 8.6	C+CKI, 20 mL, qd	XELOX	6W	①②④⑥⑦⑧⑨⑩
Tong et al. (2018)	30	30	18/12	20/10	47.67 ± 7.31	47.51 ± 7.23	C+CKI, 20 mL, qd	XELOX	4W	①②③④⑤⑥⑦⑩
Wang (2020)	49	49	28/21	31/18	56.17 ± 5.36	56.09 ± 5.18	C+CKI, 25 mL, bid	FOLFOX4	8W	②③④⑤
Wang et al. (2021)	30	30	15/15	17/13	42.51 ± 6.59	43.87 ± 7.03	C+CKI, 20 mL, qd	FOLFOX4	6W	②③④⑥⑦
Wang Z. et al., 2024	42	41	25/17	23/18	53.8 ± 10.3	51.8 ± 10.8	C+CKI, 20 mL, qd	FOLFOX4	8W	①②③④⑥⑦⑩
Xue (2021)	41	41	23/18	24/17	56.61 ± 7.09	57.24 ± 6.39	C+CKI, 15 mL, qd	Raltitrexed + Oxaliplatin	6W	②③④⑥⑦⑩
Yin et al. (2020)	68	68	33/35	30/38	53.9 ± 4.0	54.2 ± 3.5	C+CKI, 12 mL, qd	XELOX	12W	①②③④⑤
Yuan (2016)	40	43	24/16	26/17	52.26 ± 3.58	53.69 ± 4.19	C+CKI, 20 mL, qd	FOLFOX4	8W	②③④⑥⑦⑩
Zhang and Bai (2017)	23	23	15/8	13/10	48.67 ± 9. 12	49.68 ± 8. 41	C+CKI, 20 mL, qd	Oxaliplatin + 5-FU	8W	①②④⑤⑥⑦⑩
Zhang and Wu (2017)	41	41	22/19	27/14	63.1 ± 7.5	63.9 ± 7.1	C+CKI, 20 mL, qd	FOLFOX4	8W	①②③④⑥⑦⑩
Zhang et al. (2019)	58	61	31/27	35/26	52.23 ± 3.39	50.91 ± 5.08	C+CKI, 12 mL, bid	FOLFOX4	8W	①②③④⑤⑥⑦
Zhang D. W. et al., 2023	40	40	23/17	24/16	59.48 ± 10.04	59.05 ± 11.07	C+CKI, 20 mL, qd	XELOX	6W	①②③④⑤⑥⑦⑩
Zhao et al. (2022)	32	32	19/13	18/14	56.85 ± 6.05	56.87 ± 5.78	C+CKI, 12 mL, qd	FOLFOX4	4W	②③④⑤⑥⑦⑩
Zheng et al. (2011)	36	20	40/16		55.4		C+CKI, 15 mL, qd	FOLFOX4	10d	①②③④⑥⑦
Zhou et al. (2024)	37	37	23/14	24/13	58.2		C+CKI, 20 mL, qd	Bevacizumab + XELOX	9W	①②③④⑥⑦⑧⑨⑩

Abbreviations: C, control group; T, treatment group; M, male; F, female; qd, quaque in die; bid, bis in die; CKI, Compound Kushen Injection; FOLFOX4, Oxaliplatin + Fluorouracil + Folinic acid; XELOX, Oxaliplatin + Capecitabine; RALOX, Oxaliplatin + Raltitrexed; DP, Cisplatin + Docetaxel; 5-FU, Fluorouracil; Outcomes: ①CD3+; ②CD4+; ③CD8+; ④CD4+/CD8+; ⑤NK cell; ⑥ORR; ⑦DCR; ⑧CEA;⑨CA199;⑩Adverse reactions.

### 3.2 Risk of bias assessment

**FIGURE 2:**
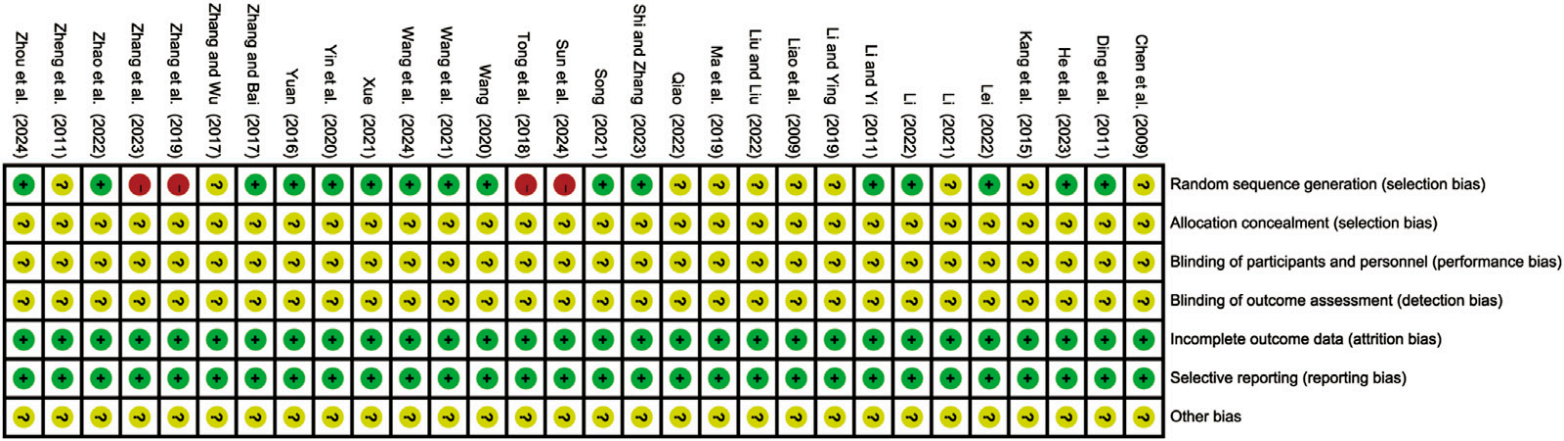
Bias risk assessment of included studies.

Baseline comparability between groups was reported in all studies. Among them, 16 studies (Ding et al., 2010; He et al., 2023; Lei, 2022; Li, 2022; Li and Yi, 2011; Shi and Zhang, 2023; Song, 2021; Wang et al., 2021; Wang Z. et al., 2024; Wang, 2020; Xue, 2021; Yin et al., 2020; Yuan, 2016; Zhang and Bai, 2017; Zhao et al., 2022; Zhou et al., 2024) were rated as low risk because they explicitly used random number tables for group allocation. Four studies (Sun et al., 2024; Tong et al., 2018; Zhang et al., 2019; Zhang D. W. et al., 2023) were assessed as high risk: three allocated participants based on treatment methods, and one grouped participant using hospital admission numbers. The remaining studies (Chen et al., 2009; Kang et al., 2015; Li and Ying, 2019; Li, 2021; Liao et al., 2009; Liu and Liu, 2022; Ma et al., 2019; Qiao, 2022; Zhang and Wu, 2017; Zheng et al., 2011) did not specify the method of randomization and were therefore classified as unclear. None of the studies reported using blinding or allocation concealment, leading to an unclear risk rating for these domains. All studies reported no loss to follow-up, resulting in a low risk assessment for the domain of completeness of outcome data. Additionally, outcomes were clearly defined and comprehensively reported in all studies, resulting in a low risk assessment for selective reporting. No study explicitly identified other sources of bias, leading to an overall unclear risk rating for other biases. Overall, the quality of the included studies was relatively low. The results of the risk of bias assessment are presented in Figure 2.

### 3.3 Primary outcomes

#### 3.3.1 CD3^+^ levels

**FIGURE3:**
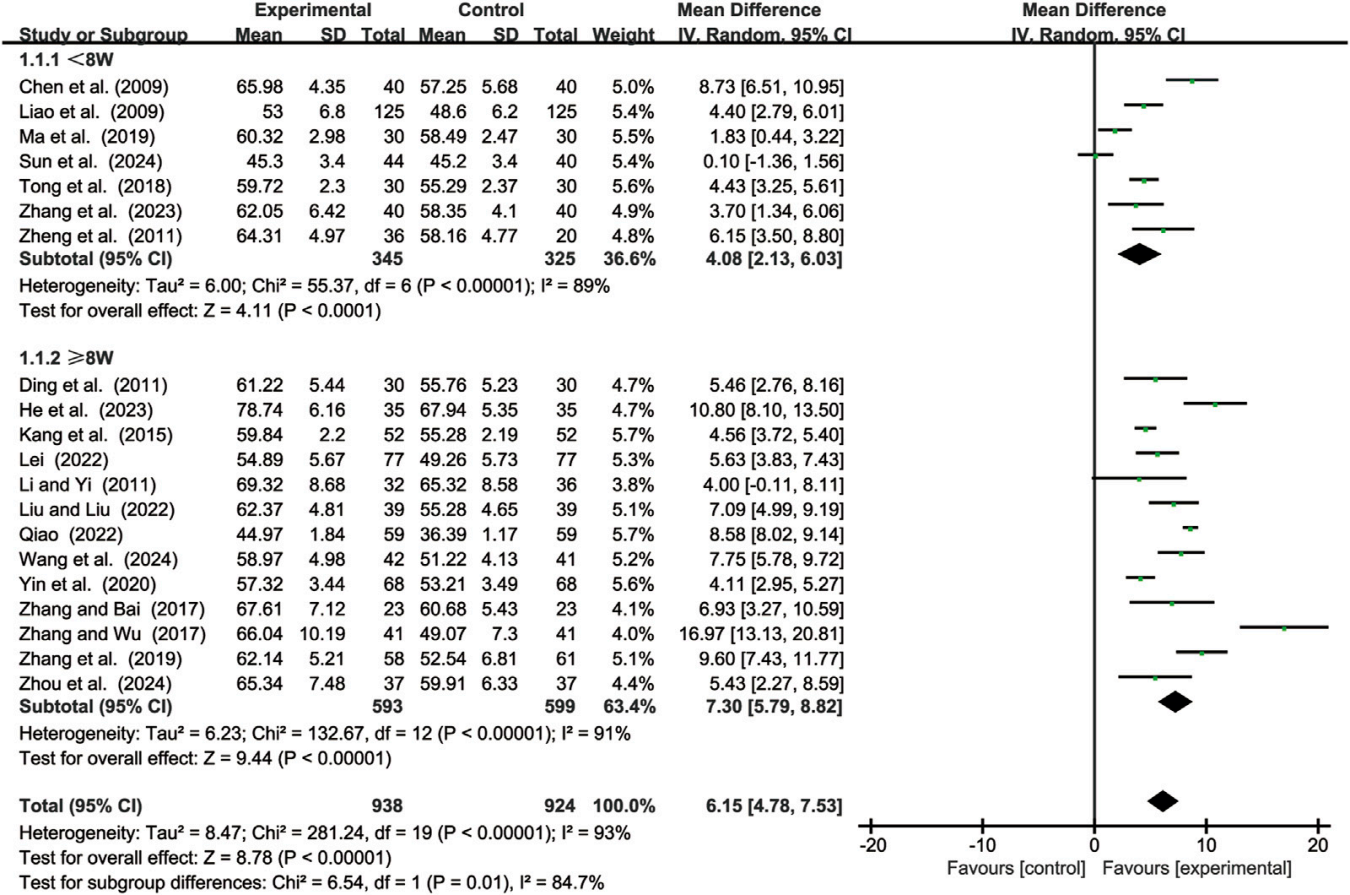
Forest plot for CD3^+^ levels.

20 studies (Chen et al., 2009; Ding et al., 2010; He et al., 2023; Kang et al., 2015; Lei, 2022; Li and Yi, 2011; Liao et al., 2009; Liu and Liu, 2022; Ma et al., 2019; Qiao, 2022; Sun et al., 2024; Tong et al., 2018; Wang Z. et al., 2024; Yin et al., 2020; Zhang et al., 2019; Zhang D. W. et al., 2023; Zhang and Wu, 2017; Zhang and Bai, 2017; Zheng et al., 2011; Zhou et al., 2024) evaluated CD3^+^ levels. Due to significant heterogeneity (*p* < 0.00001, *I* ^2^ = 93%), a random-effects model was used to pool the effect sizes. CKI significantly improved CD3^+^ levels compared with CC alone [MD = 6.15, 95% CI: 4.78 to 7.53, *p* < 0.00001, Figure 3]. Subgroup analysis based on treatment duration revealed significant improvements between CKI and CC for both durations: less than 8 weeks [MD = 4.08, 95% CI: 2.13 to 6.03, *p* < 0.0001, Figure 3] and 8 weeks or longer [MD = 7.30, 95% CI: 5.79 to 8.82, *p* < 0.00001, Figure 3].

#### 3.3.2 CD4^+^ levels

**FIGURE 4:**
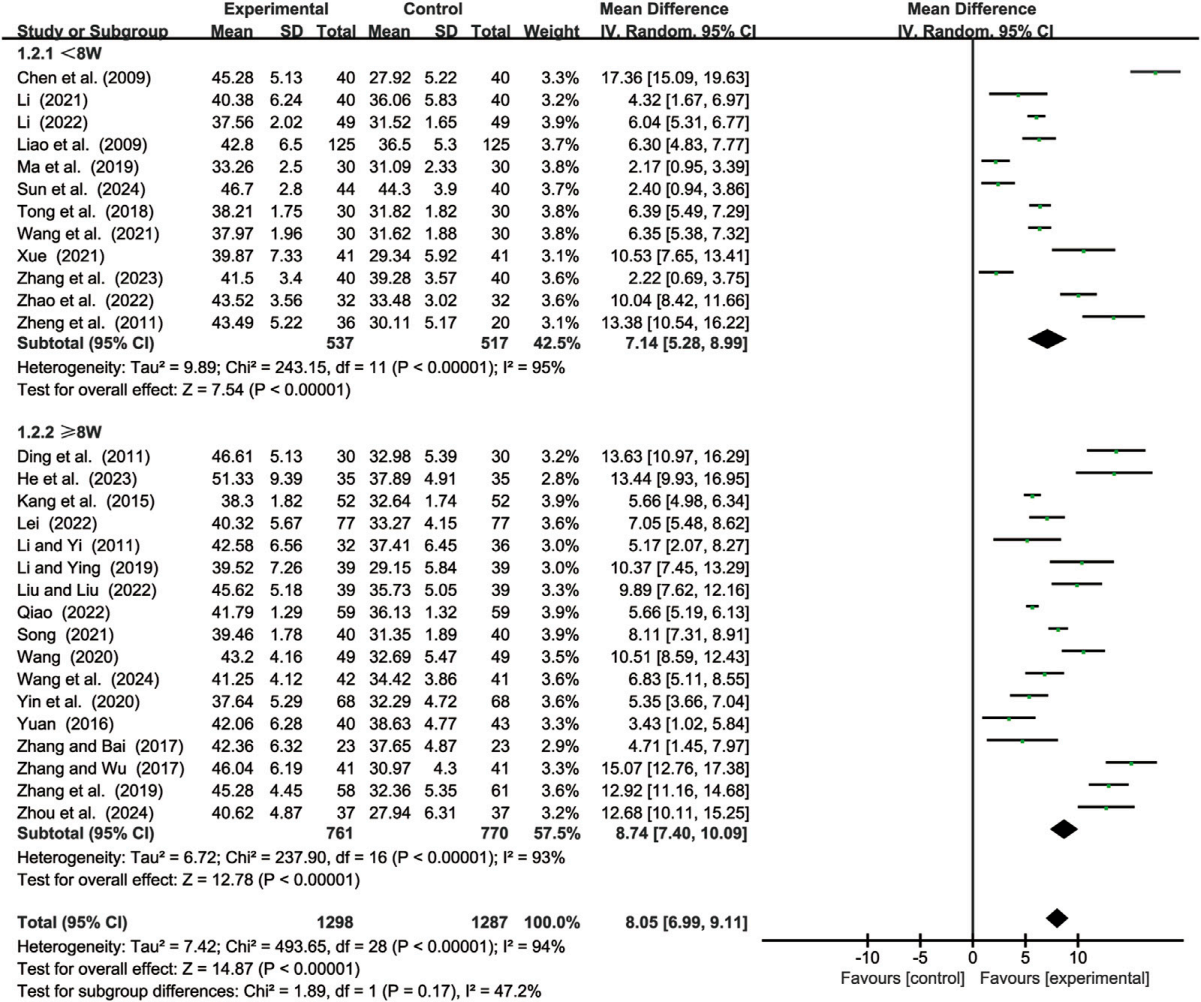
Forest plot for CD4^+^ levels.

29 studies (Chen et al., 2009; Ding et al., 2010; He et al., 2023; Kang et al., 2015; Lei, 2022; Li and Ying, 2019; Li, 2022; Li and Yi, 2011; Li, 2021; Liao et al., 2009; Liu and Liu, 2022; Ma et al., 2019; Qiao, 2022; Song, 2021; Sun et al., 2024; Tong et al., 2018; Wang et al., 2021; Wang Z. et al., 2024; Wang, 2020; Xue, 2021; Yin et al., 2020; Yuan, 2016; Zhang et al., 2019; Zhang D. W. et al., 2023; Zhang and Wu, 2017; Zhang and Bai, 2017; Zhao et al., 2022; Zheng et al., 2011; Zhou et al., 2024) evaluated CD4^+^ levels. Due to significant heterogeneity (*p* < 0.00001, *I* ^2^ = 94%), a random-effects model was used to pool the effect sizes. CKI significantly improved CD4^+^ levels compared with CC alone [MD = 8.05, 95% CI: 6.99 to 9.11, *p* < 0.00001, Figure 4]. Subgroup analysis based on treatment duration revealed significant improvements between CKI and CC for both durations: less than 8 weeks [MD = 7.14, 95% CI: 5.28 to 8.99, *p* < 0.0001, Figure 4] and 8 weeks or longer [MD = 8.74, 95% CI: 7.40 to 10.09, *p* < 0.00001, Figure 4].

#### 3.3.3 CD8^+^ levels

**FIGURE 5:**
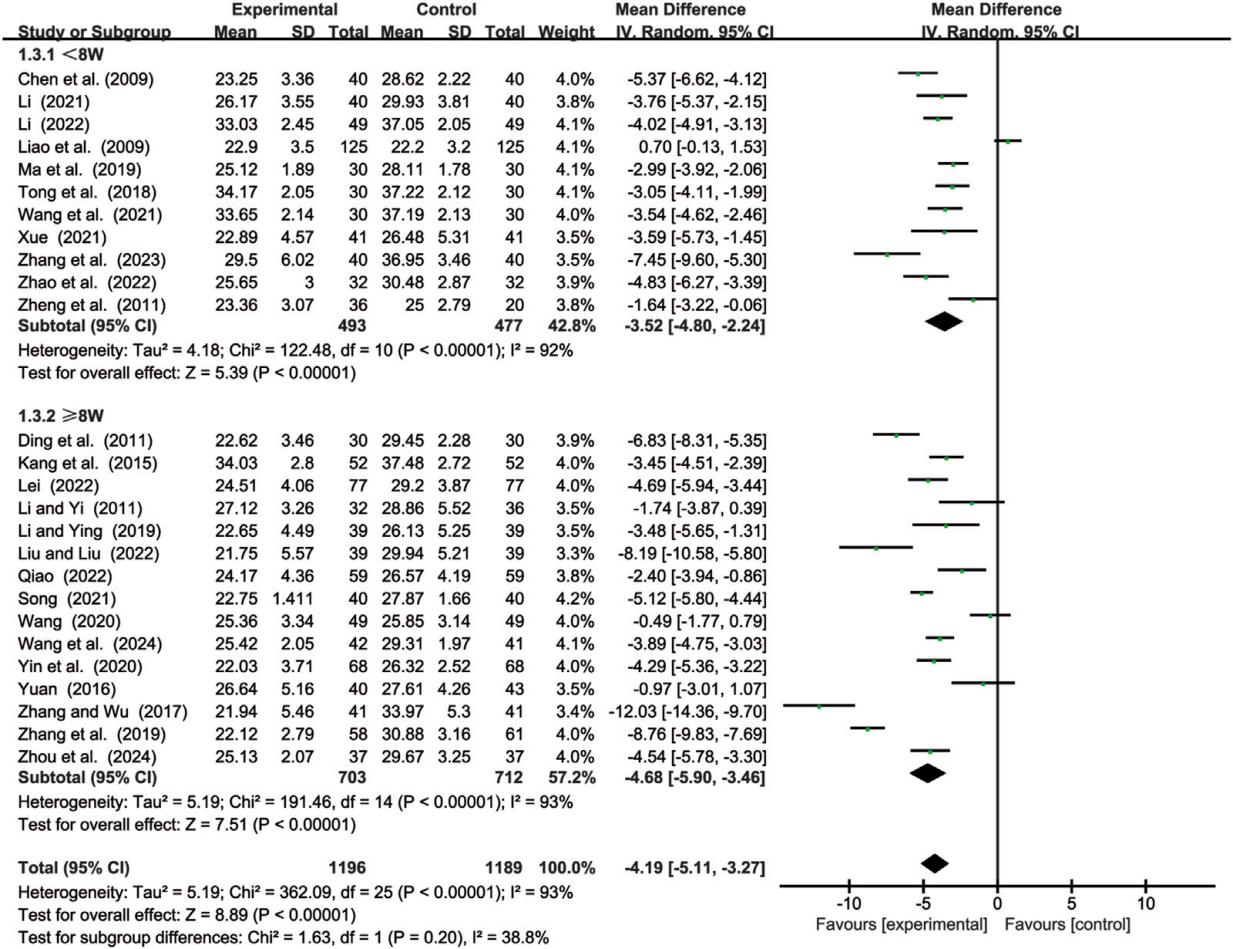
Forest plot for CD8^+^ levels.

26 studies (Chen et al., 2009; Ding et al., 2010; Kang et al., 2015; Lei, 2022; Li and Ying, 2019; Li, 2022; Li and Yi, 2011; Li, 2021; Liao et al., 2009; Liu and Liu, 2022; Ma et al., 2019; Qiao, 2022; Song, 2021; Tong et al., 2018; Wang et al., 2021; Wang Z. et al., 2024; Wang, 2020; Xue, 2021; Yin et al., 2020; Yuan, 2016; Zhang et al., 2019; Zhang D. W. et al., 2023; Zhang and Wu, 2017; Zhao et al., 2022; Zheng et al., 2011; Zhou et al., 2024) evaluated CD8^+^ levels. Due to significant heterogeneity (*p* < 0.00001, *I* ^2^ = 93%), a random-effects model was used to pool the effect sizes. CKI significantly reduced CD8^+^ levels compared with CC alone [MD = −4.19, 95% CI: −5.11 to −3.27, *p* < 0.00001, Figure 5]. Subgroup analysis based on treatment duration revealed significant reductions between CKI and CC for both durations: less than 8 weeks [MD = −3.52, 95% CI: −4.80 to −2.24, *p* < 0.00001, Figure 5] and 8 weeks or longer [MD = −4.68, 95% CI: −5.90 to −3.46, *p* < 0.00001, Figure 5].

#### 3.3.4 CD4+/CD8+ levels

**FIGURE 6:**
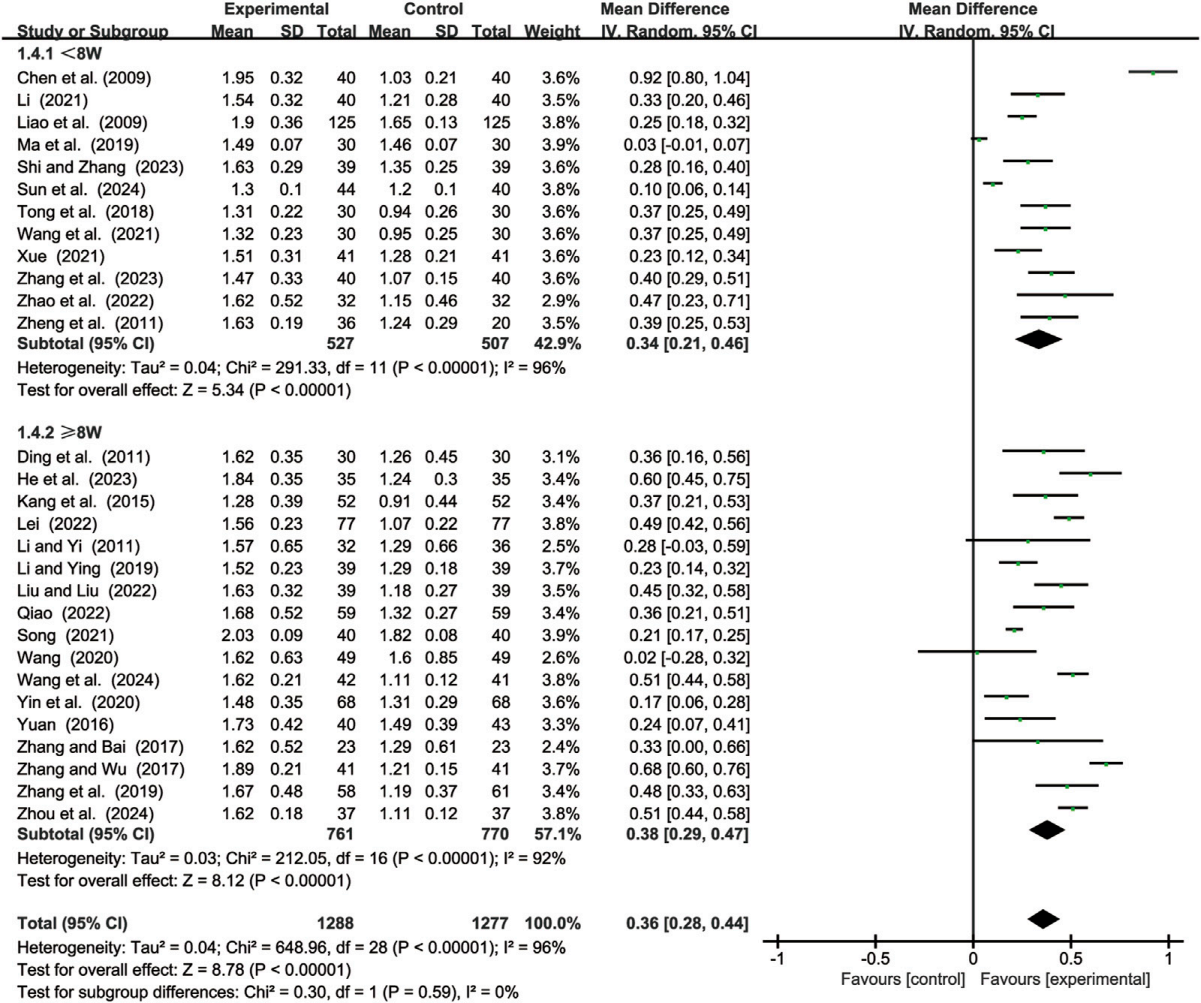
Forest plot for CD4+/CD8+ levels.

29 studies (Chen et al., 2009; Ding et al., 2010; He et al., 2023; Kang et al., 2015; Lei, 2022; Li and Ying, 2019; Li and Yi, 2011; Li, 2021; Liao et al., 2009; Liu and Liu, 2022; Ma et al., 2019; Qiao, 2022; Shi and Zhang, 2023; Song, 2021; Sun et al., 2024; Tong et al., 2018; Wang et al., 2021; Wang Z. et al., 2024; Wang, 2020; Xue, 2021; Yin et al., 2020; Yuan, 2016; Zhang et al., 2019; Zhang D. W. et al., 2023; Zhang and Wu, 2017; Zhang and Bai, 2017; Zhao et al., 2022; Zheng et al., 2011; Zhou et al., 2024) evaluated CD4/CD8+ levels. Due to significant heterogeneity (*p* < 0.00001, *I* ^2^ = 96%), a random-effects model was used to pool the effect sizes. CKI significantly improved CD4+/CD8+ levels compared with CC alone [MD = 0.36, 95% CI: 0.28 to 0.44, *p* < 0.00001, Figure 6]. Subgroup analysis based on treatment duration revealed significant improvements between CKI and CC for both durations: less than 8 weeks [MD = 0.34, 95% CI: 0.21 to 0.46, *p* < 0.00001, Figure 6] and 8 weeks or longer [MD = 0.38, 95% CI: 0.29 to 0.47, *p* < 0.00001, Figure 6].

#### 3.3.5 NK cell levels

**FIGURE 7:**
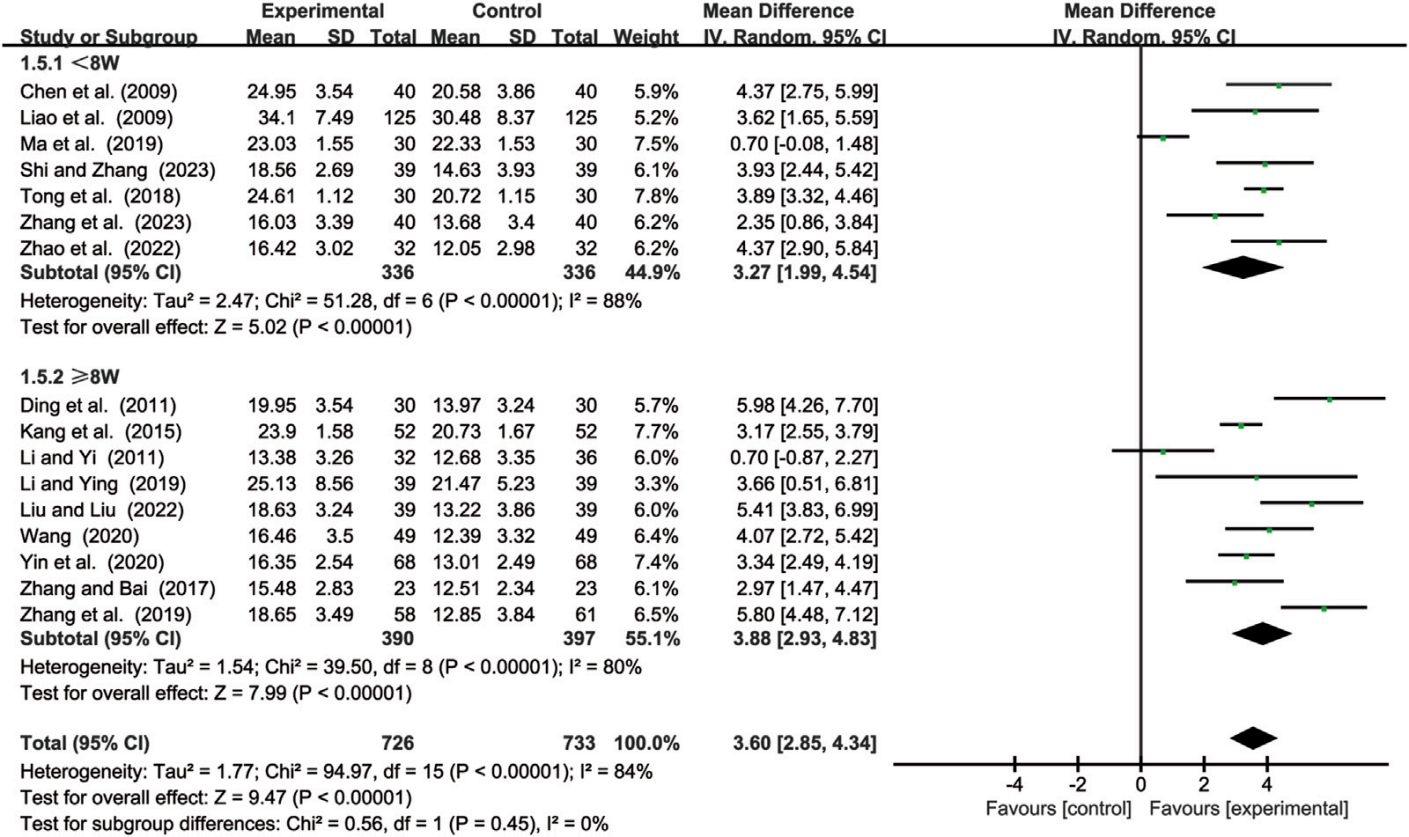
Forest plot for NK cell levels.

**FIGURE 8:**
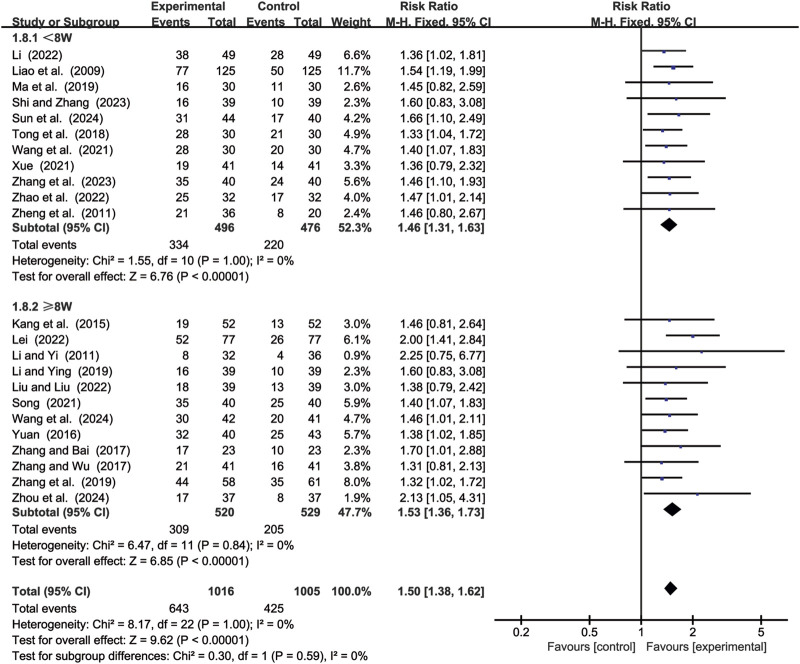
Forest plot for ORR.

16 studies (Chen et al., 2009; Ding et al., 2010; Kang et al., 2015; Li and Ying, 2019; Li and Yi, 2011; Liao et al., 2009; Liu and Liu, 2022; Ma et al., 2019; Shi and Zhang, 2023; Tong et al., 2018; Wang, 2020; Yin et al., 2020; Zhang et al., 2019; Zhang D. W. et al., 2023; Zhang and Bai, 2017; Zhao et al., 2022) evaluated NK cell levels. Due to significant heterogeneity (*p* < 0.00001, *I* ^2^ = 84%), a random-effects model was used to pool the effect sizes. CKI significantly improved NK cell levels compared with CC alone [MD = 3.60, 95% CI: 2.85 to 4.34, *p* < 0.00001, Figure 7]. Subgroup analysis based on treatment duration revealed significant improvements between CKI and CC for both durations: less than 8 weeks [MD = 3.27, 95% CI: 1.99 to 4.54, *p* < 0.00001, Figure 7] and 8 weeks or longer [MD = 3.88, 95% CI: 2.93 to 4.83, *p* < 0.00001, Figure 7].

### 3.4 Secondary outcomes

#### 3.4.1 ORR

23 studies (Kang et al., 2015; Lei, 2022; Li and Ying, 2019; Li, 2022; Li and Yi, 2011; Liao et al., 2009; Liu and Liu, 2022; Ma et al., 2019; Shi and Zhang, 2023; Song, 2021; Sun et al., 2024; Tong et al., 2018; Wang et al., 2021; Wang Z. et al., 2024; Xue, 2021; Yuan, 2016; Zhang et al., 2019; Zhang D. W. et al., 2023; Zhang and Wu, 2017; Zhang and Bai, 2017; Zhao et al., 2022; Zheng et al., 2011; Zhou et al., 2024) evaluated ORR. Due to low heterogeneity (*p* = 1.00, *I* ^2^ = 0%), a fixed-effects model was used to pool the effect sizes. CKI significantly improved ORR compared with CC alone [RR = 1.50, 95% CI: 1.38 to 1.62, *p* < 0.00001, Figure 8]. Subgroup analysis based on treatment duration revealed significant improvements between CKI and CC for both durations: less than 8 weeks [RR = 1.46, 95% CI: 1.31 to 1.63, *p* < 0.00001, Figure 8] and 8 weeks or longer [RR = 1.53, 95% CI: 1.36 to 1.73, *p* < 0.00001, Figure 8].

#### 3.4.2 DCR

**FIGURE 9:**
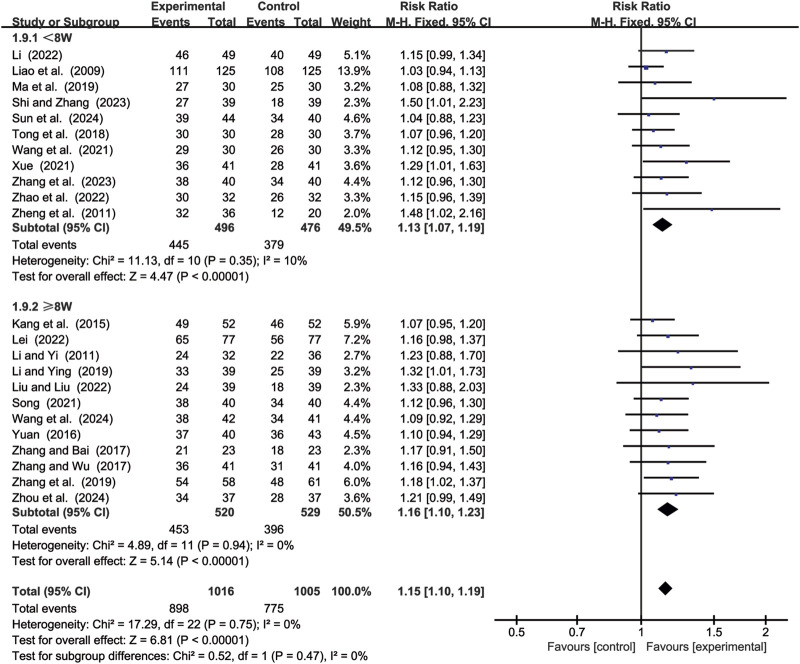
Forest plot for DCR.

23 studies (Kang et al., 2015; Lei, 2022; Li and Ying, 2019; Li, 2022; Li and Yi, 2011; Liao et al., 2009; Liu and Liu, 2022; Ma et al., 2019; Shi and Zhang, 2023; Song, 2021; Sun et al., 2024; Tong et al., 2018; Wang et al., 2021; Wang Z. et al., 2024; Xue, 2021; Yuan, 2016; Zhang et al., 2019; Zhang D. W. et al., 2023; Zhang and Wu, 2017; Zhang and Bai, 2017; Zhao et al., 2022; Zheng et al., 2011; Zhou et al., 2024) evaluated DCR. Due to low heterogeneity (*p* = 0.75, *I* ^2^ = 0%), a fixed-effects model was used to pool the effect sizes. CKI significantly improved DCR compared with CC alone [RR = 1.15, 95% CI: 1.10 to 1.19, *p* < 0.00001, Figure 9]. Subgroup analysis based on treatment duration revealed significant improvements between CKI and CC for both durations: less than 8 weeks [RR = 1.13, 95% CI: 1.07 to 1.19, *p* < 0.00001, Figure 9] and 8 weeks or longer [RR = 1.16, 95% CI: 1.10 to 1.23, *p* < 0.00001, Figure 9].

#### 3.4.3 CEA levels

**FIGURE 10:**
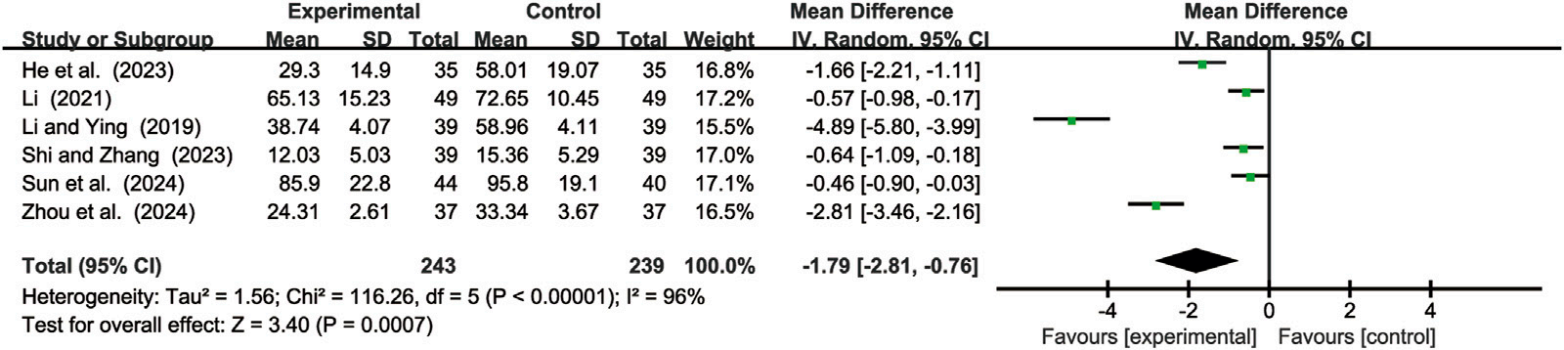
Forest plot for CEA levels.

Six studies (He et al., 2023; Li and Ying, 2019; Li, 2022; Shi and Zhang, 2023; Sun et al., 2024; Zhou et al., 2024) evaluated CEA levels. Due to significant heterogeneity (*p* < 0.00001, *I* ^2^ = 96%), a random-effects model was used to pool the effect sizes. CKI significantly reduced CEA levels compared with CC alone [MD = −1.79, 95% CI: −2.81 to −0.76, *p* = 0.0007, Figure 10].

#### 3.4.4 CA199 levels

**FIGURE 11:**
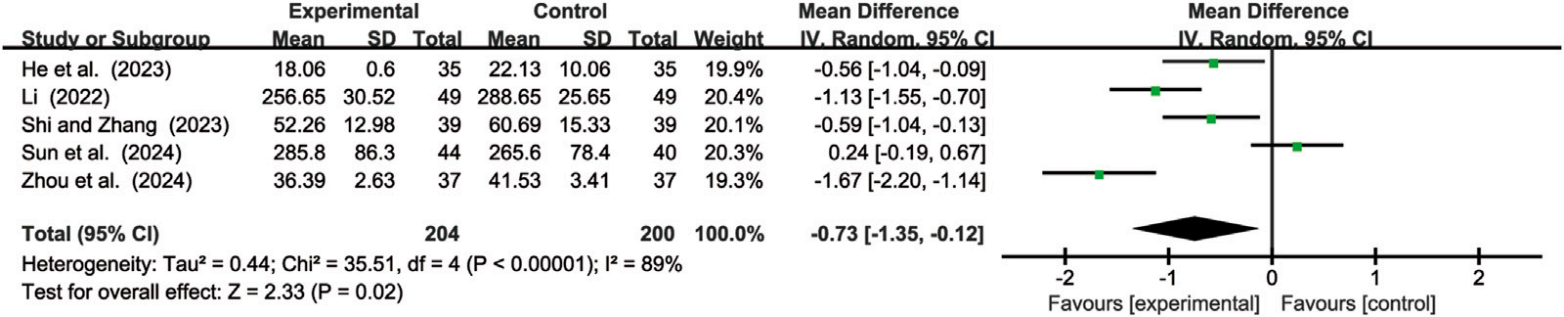
Forest plot for CA199 levels.

Five studies (He et al., 2023; Li, 2022; Shi and Zhang, 2023; Sun et al., 2024; Zhou et al., 2024) evaluated CA199 levels. Due to significant heterogeneity (*p* < 0.00001, *I* ^2^ = 89%), a random-effects model was used to pool the effect sizes. CKI significantly reduced CA199 levels compared with CC alone [MD = −0.73, 95% CI: −1.35 to −0.12, *p* = 0.02, Figure 11].

#### 3.4.5 Adverse reactions

The adverse reactions of CKI in treating CRC included nausea and vomiting, hepatic dysfunction, myelosuppression, neurotoxicity, leukopenia, thrombocytopenia, and mouth ulcer. CKI significantly reduced chemotherapy-induced adverse reactions compared with CC alone: nausea and vomiting [RR = 0.62, 95% CI: 0.54 to 0.71, *p* < 0.00001], hepatic dysfunction [RR = 0.49, 95% CI: 0.40 to 0.61, *p* < 0.00001], myelosuppression [RR = 0.63, 95% CI: 0.54 to 0.74, *p* < 0.00001], neurotoxicity [RR = 0.64, 95% CI: 0.49 to 0.82, *p* = 0.0006], leukopenia [RR = 0.61, 95% CI: 0.49 to 0.76, *p* < 0.0001], thrombocytopenia [RR = 0.55, 95% CI: 0.34 to 0.90, *p* = 0.02], and mouth ulcers [RR = 0.59, 95% CI: 0.38 to 0.93, *p* = 0.02]. The details are presented in Table 2.

**TABLE 2:** Adverse reactions of the included studies.

Adverse reactions	Included studies	Treatment group		Control group		Heterogeneity		Outcomes	
		Events	Total	Events	Total	*I* ^2^ (%)	*P*	RR (M-H, Fixed, 95% CI)	*P*
Nausea and vomiting	He et al. (2023); Lei (2022); Li (2021); Li (2022); Li and Ying (2019); Liu and Liu (2022); Ma et al. (2019); Qiao (2022); Shi and Zhang (2023); Song (2021); Sun et al. (2024); Tong et al. (2018); Wang Z. et al., 2024; Xue (2021); Yuan (2016); Zhang and Bai (2017); Zhang and Wu (2017); Zhang D. W. et al., 2023; Zhao et al. (2022); Zhou et al. (2024)	200	817	321	815	13	0.29	0.62 (0.54, 0.71)	<0.00001
Hepatic dysfunction	He et al. (2023); Lei (2022); Li (2022); Li and Ying (2019); Shi and Zhang (2023); Song (2021); Wang Z. et al., 2024	85	534	171	536	0	0.93	0.49 (0.40, 0.61)	<0.00001
Myelosuppression	He et al. (2023); Li (2022); Li and Ying (2019); Liu and Liu (2022); Ma et al. (2019); Sun et al. (2024); Tong et al. (2018); Wang Z. et al., 2024; Xue (2021); Zhang D. W. et al., 2023; Zhao et al. (2022)	124	421	191	416	0	0.56	0.63 (0.54, 0.74)	<0.00001
Neurotoxicity	Lei (2022); Li (2021); Liu and Liu (2022); Qiao (2022); Wang Z. et al., 2024; Xue (2021); Yuan (2016); Zhang D. W. et al., 2023; Zhou et al. (2024)	66	415	104	417	0	0.90	0.64 (0.49, 0.82)	0.0006
Leukopenia	Lei (2022); Li (2021); Shi and Zhang (2023); Song (2021); Tong et al. (2018); Yuan (2016); Zhang and Bai (2017); Zhang and Wu (2017); Zhou et al. (2024)	82	367	135	370	34	0.15	0.61 (0.49, 0.76)	<0.0001
Thrombocytopenia	Lei (2022); Shi and Zhang (2023); Zhang and Bai (2017); Zhang and Wu (2017)	21	180	38	180	0	1.00	0.55 (0.34, 0.90)	0.02
Mouth ulcer	Lei (2022); Ma et al. (2019); Tong et al. (2018); Zhang and Bai (2017)	23	160	39	160	0	0.77	0.59 (0.38, 0.93)	0.02

### 3.5 Sensitivity analysis

**FIGURE 12:**
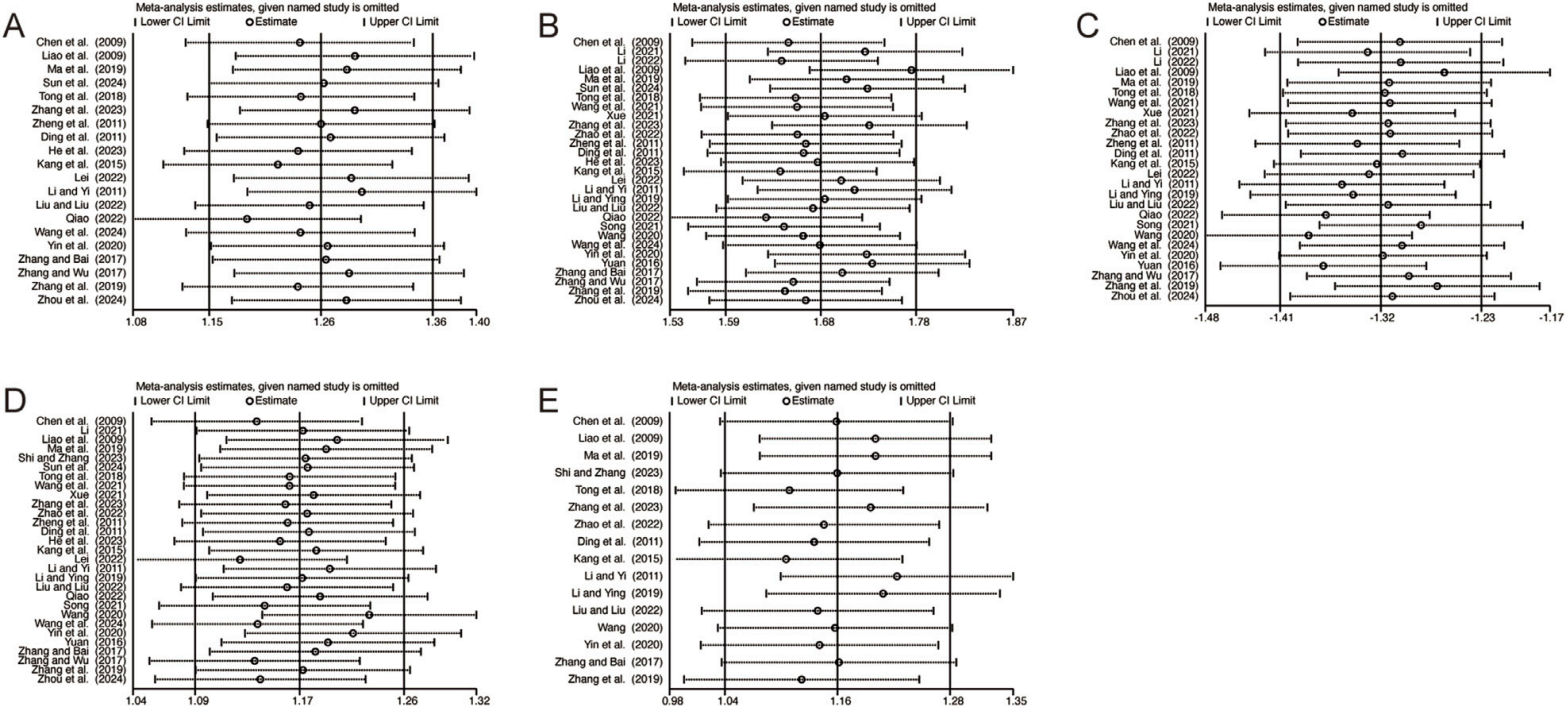
The results of sensitivity analysis. **(A)** CD3^+^ levels. **(B)** CD4^+^ levels. **(C)** CD8^+^ levels. **(D)** CD4+/CD8+ levels. **(E)** NK cell levels.

To evaluate the robustness and reliability of the results, sensitivity analyses were performed for the primary outcomes, including CD3^+^, CD4^+^, CD8^+^, CD4+/CD8+, and NK cell levels, by sequentially excluding individual studies. This approach assessed the influence of each study on the pooled results. The findings demonstrated that no single study exerted a significant impact on the combined results, thereby confirming the robustness and reliability of the outcomes (Figure 12).

### 3.6 Publication bias

**FIGURE 13:**
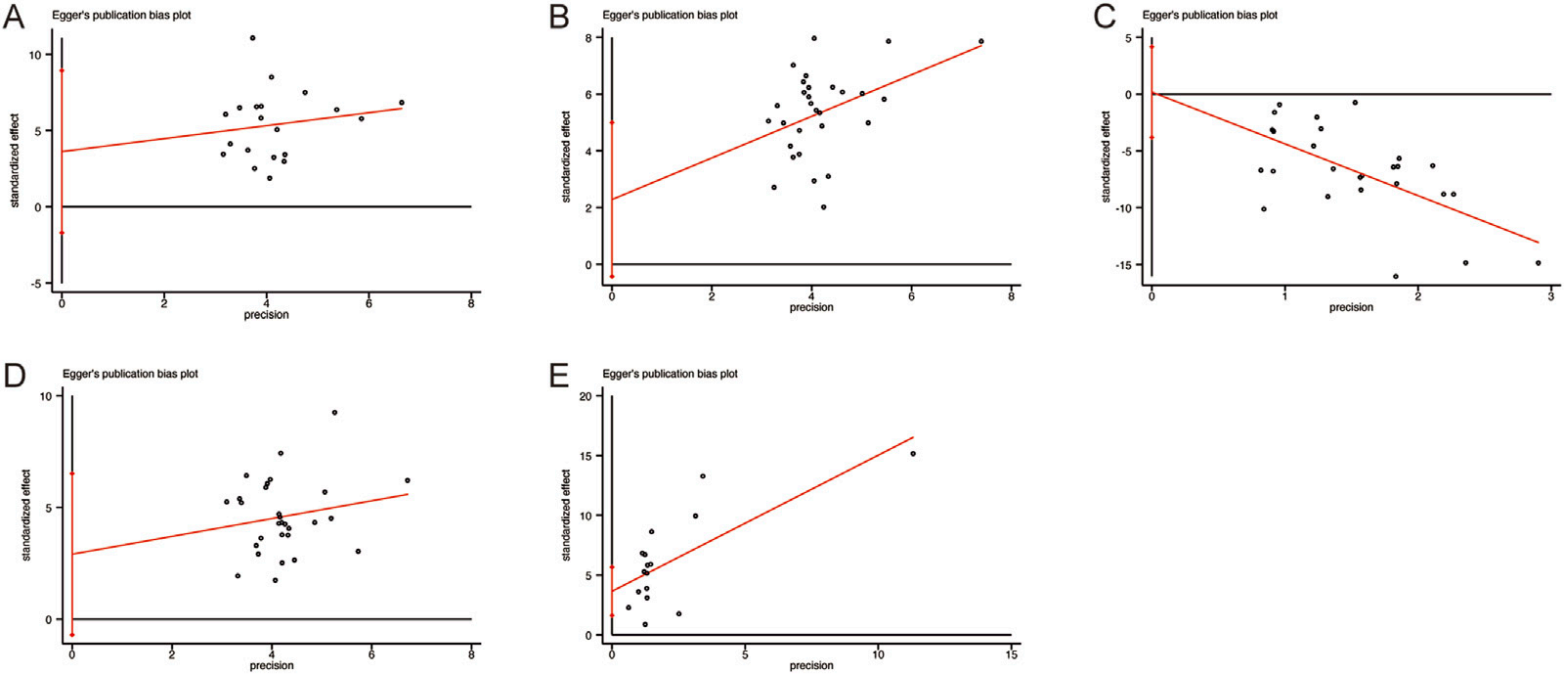
Egger’s publication funnel plot. **(A)** CD3^+^ levels. **(B)** CD4^+^ levels. **(C)** CD8^+^ levels. **(D)** CD4+/CD8+ levels. **(E)** NK cell levels.

Given the substantial heterogeneity observed in the primary outcomes, Egger’s test was conducted to assess potential publication bias (Figure 13). The results showed no significant publication bias for any of the primary outcomes: CD3^+^ levels (*p* = 0.170), CD4^+^ levels (*p* = 0.095), CD8^+^ levels (*p* = 0.928), CD4+/CD8+ levels (*p* = 0.111), and NK cell levels (*p* = 0.173). These findings suggest that the results were not significantly influenced by publication bias.

## 4 Discussion

CRC is among the most prevalent malignancies worldwide, ranking third in incidence and second in cancer-related mortality (Sung et al., 2021). Early-stage CRC often presents with nonspecific symptoms, such as dyspepsia or occult blood in stool, and may progress to more severe manifestations, including abdominal pain, rectal bleeding, and intestinal obstruction (Li et al., 2024). Surgical resection remains the primary treatment for early to middle-stage CRC, whereas advanced and recurrent cases require adjuvant therapies such as chemotherapy and radiotherapy (Van Cutsem et al., 2016). Postoperative chemotherapy, particularly oxaliplatin-based regimens, is critical for preventing recurrence and metastasis (Hong et al., 2019). However, such treatments are often associated with severe adverse reactions, including myelosuppression, gastrointestinal reactions, and immune dysfunction, which significantly impair patients’ quality of life and may lead to discontinuation of therapy, reducing clinical efficacy (Moisuc et al., 2023). Therefore, identifying effective and less toxic combination therapies is an urgent priority in CRC management.

CKI, a traditional Chinese medicine preparation, has shown promise in mitigating chemotherapy-induced toxicity, enhancing antitumor efficacy, and regulating immune function (Dong et al., 2019). Pharmacological studies have demonstrated that its key components, such as matrine and oxymatrine, exert antitumor effects through various mechanisms, including apoptosis induction, cell cycle arrest, and inhibition of key signaling pathways such as Wnt/β-catenin and PI3K/AKT/mTOR (Chen et al., 2021; Halim et al., 2019; Zhang and Shen, 2020). Furthermore, CKI has been shown to alleviate chemotherapy side effects, enhance NK cell activity, and improve patients’ quality of life (Chen et al., 2018). Given the critical role of immune function in CRC progression, this meta-analysis aims to systematically evaluate the impact of CKI on immune function in CRC patients, providing evidence to guide clinical decision-making.

### 4.1 Summary of findings

This meta-analysis is the first to assess the impact of CKI on immune function in CRC patients. Key findings include: (a) CKI combined with CC significantly improved immune function, as evidenced by increased levels of CD3^+^, CD4^+^, CD4+/CD8+, and NK cell, along with decreased CD8^+^ levels. (b) CKI demonstrated superior ORR and DCR compared to CC alone, with reductions in CEA and CA199 levels. (c) CKI reduced chemotherapy-induced adverse reactions, including nausea, vomiting, liver dysfunction, myelosuppression, neurotoxicity, and leukopenia. Sensitivity analysis confirmed the robustness of these findings, and Egger’s test indicated no significant publication bias. Overall, this analysis highlights CKI’s potential to enhance immune function, improve clinical outcomes, and mitigate adverse reactions in CRC patients.

### 4.2 Comparison with previous studies

A prior meta-analysis by Wu et al. (2024) examined the clinical efficacy and adverse reactions of CKI in CRC treatment but did not comprehensively evaluate its impact on immune function. Limitations of earlier research include small sample sizes, lack of consideration for treatment duration, and reliance on funnel plots to assess publication bias, which may undermine result reliability. Additionally, previous studies primarily focused on clinical outcomes, while the immunomodulatory effects of CKI—a critical factor in CRC progression and treatment response—were not systematically analyzed. This study addresses these gaps by conducting a more detailed evaluation of CKI’s effects on immune function, specifically assessing CD4^+^, CD8^+^, and CD4+/CD8+ ratios, and linking these immune parameters to ORR and DCR. Furthermore, we incorporated subgroup analyses to examine the influence of treatment duration on both immune modulation and overall therapeutic benefits. By refining methodological approaches and expanding the scope of analysis, this study provides stronger evidence for CKI’s therapeutic potential, offering valuable insights into its immunoregulatory role in CRC management.

### 4.3 Mechanistic insights of CKI on immune modulation

Mechanistically, CKI has been shown to modulate immune function through multiple pathways. Single-cell RNA sequencing and transcriptome analyses have demonstrated that CKI enhances immune cell infiltration into the tumor microenvironment, particularly increasing CD8^+^ T cell activation and NK cell cytotoxicity (Liu et al., 2022; Liu et al., 2021). Additionally, CKI has been reported to relieve tumor-associated macrophage-mediated immunosuppression in hepatocellular carcinoma by triggering TNFR1-mediated NF-κB and p38 MAPK signaling, subsequently improving CD8^+^ T cell cytotoxicity and tumor clearance (Yang et al., 2020). In breast cancer models, CKI has been found to enhance the anti-tumor effects of chemotherapy by upregulating IL-1β and modulating immune-related pathways, further supporting its potential immunotherapeutic role (Shen et al., 2019). Recent bioinformatics analyses also suggest that oxymatrine, one of CKI’s key bioactive components, may regulate immune response through the TGF-β/Smad and Wnt signaling pathways (Jin et al., 2024). These mechanistic insights align with the findings of this meta-analysis, which showed increased CD3^+^, CD4^+^, and NK cell levels following CKI treatment, along with a reduction in CD8^+^ levels. The decrease in CD8^+^ T cells may reflect the removal of ineffective or exhausted subsets, optimizing immune balance. However, CKI’s precise effects on CD8^+^ T cell subsets remain unclear. Future studies should use flow cytometry to distinguish effector and exhausted CD8^+^ T cells, clarifying CKI’s immunoregulatory role in CRC treatment.

### 4.4 Strengths and limitations

CRC progression and recurrence are closely linked to immune dysfunction, with T cell levels serving as critical indicators of immune status (Pardoll, 2012). CD3^+^ T cells mediate immune responses and are capable of killing tumor cells (Shao et al., 2021). CD4^+^ T cells enhance immune responses by producing lymphokines that support other immune cells and are predictive of CRC prognosis (Wang Z. et al., 2024). CD8^+^ T cells, with their cytotoxic effects on target cells, are significant markers for evaluating postoperative outcomes in CRC patients (Li Y. et al., 2023). Additionally, the CD4+/CD8+ ratio is a key indicator of immune function and antitumor capacity in primary CRC (Tao and Xie, 2024). By focusing on these parameters, this meta-analysis offers novel insights into CKI’s role in modulating immune function in CRC patients. To improve the reliability of our findings, we performed subgroup analyses based on different treatment durations of CKI to account for potential confounding factors associated with therapy length. Furthermore, our study addresses a critical aspect of CRC management by focusing on immune dysfunction, which is a major contributor to CRC recurrence and mortality (Li et al., 2025).

Despite its contributions, this study has several limitations: (1) Variability in Treatment Regimens: The included studies differed in dosing regimens, chemotherapy protocols, and treatment durations, which may introduce potential heterogeneity and impact the results. Moreover, the specific details regarding the formulation and quality control of CKI were not consistently reported, making it difficult to assess whether differences in preparation influenced the outcomes. Additionally, CKI’s interactions with different chemotherapeutic agents remain unclear. This uncertainty could affect treatment efficacy and safety. (2) Geographical and Cultural Constraints: CKI is a traditional Chinese medicine injection approved for CRC treatment only in China. All included RCTs were conducted in Chinese clinical settings, potentially limiting the generalizability of the findings to other populations. This geographical concentration raises concerns about publication bias and cultural differences in integrative oncology practices. (3) Quality of Included Studies: Although the RCTs reported randomization, the precise methods for generating random sequences, allocation concealment, and blinding were often inadequately described. Such shortcomings increase the risk of selection, performance, and detection biases. Additionally, many studies did not provide detailed risk of bias assessments or report on adverse events in a rigorous manner, thereby affecting the overall quality and credibility of the evidence. (4) Lack of Long-Term Follow-Up: None of the included studies provided long-term follow-up data. The clinical efficacy outcomes were primarily based on short-term measures such as immune markers and response rates, without reporting overall survival (OS) or progression-free survival (PFS). As a result, the sustained impact of CKI on long-term outcomes remains unclear. (5) Unclear Impact on Tumor Microenvironment: This analysis focused on circulating immune markers without distinguishing between peripheral lymphocytes and tumor-infiltrating lymphocytes, leaving CKI’s direct impact on CRC immunity uncertain. Additionally, the lack of data on CD8^+^ cytotoxic and exhausted subpopulations makes it unclear whether its reduction reflects immune suppression or modulation. (6) Lack of Consideration for Disease Stage: CRC patients exhibit different immune profiles depending on disease stage, with advanced-stage tumors often associated with greater immune suppression. However, disease stage was not consistently reported in the included studies, making it impossible to determine whether CKI’s immunomodulatory effects differ across early- and late-stage CRC. The absence of stratification by disease stage limits the ability to interpret the findings accurately.

### 4.5 Implication

To strengthen the evidence supporting the use of CKI in the treatment of CRC, future clinical research should focus on several key areas. First, future studies should delineate the detailed mechanisms by which CKI modulates immune responses. Specifically, research must distinguish between its effects on circulating immune cells and tumor-infiltrating lymphocytes. Moreover, the observed reduction in CD8^+^ levels raises concerns about potential immune suppression. Further research should distinguish CD8^+^ subtypes (effector vs exhausted) and assess CKI’s potential synergy with immunotherapy. Second, standardization of CKI treatment protocols is essential. The included studies varied in CKI dosing regimens, chemotherapy combinations, and treatment durations, introducing potential heterogeneity. Future clinical trials should implement standardized dosing protocols and clearly define CKI formulation details, including active metabolite concentrations and quality control parameters, to enhance reproducibility and comparability across studies. Third, future RCTs should prioritize long-term outcome assessment. The current evidence is limited to short-term immune markers and response rates, with no available data on overall survival (OS) or progression-free survival (PFS). Given the importance of these endpoints in determining true clinical benefits, prospective studies with extended follow-up periods are needed to evaluate the durability of CKI’s therapeutic effects in CRC patients. Fourth, expanding the geographic and cultural scope of CKI research is necessary. Since all included studies were conducted in China, the generalizability of the findings remains uncertain. Future research should involve multicenter trials with international cohorts to assess CKI’s efficacy and safety in diverse populations. This will help determine whether its benefits extend beyond Chinese clinical settings and account for genetic or cultural differences in treatment response. Fifth, research should explore the potential synergy between CKI and emerging immunotherapies or targeted therapies, as advances in immune checkpoint inhibitors (ICIs) and molecularly targeted treatments have transformed CRC management. Future studies should investigate whether CKI enhances ICI efficacy (e.g., anti-PD-1/PD-L1 therapy) or mitigates immune-related adverse effects. Additionally, mechanistic studies should assess CKI’s interactions with chemotherapy, as it may enhance some agents while antagonizing others, such as 5-FU. Pharmacokinetic and pharmacodynamic research is needed to determine optimal combination strategies and their impact on efficacy and toxicity, ensuring safe and effective integration of CKI into multimodal treatment regimens. Notably, an ongoing multicenter RCT (NCT05894694) is evaluating CKI combined with first-line chemotherapy for advanced CRC, with PFS as the primary endpoint. Its findings will provide critical insights into CKI’s role in advanced disease and may guide biomarker-driven patient selection. Sixth, future studies should investigate the impact of disease stage on CKI’s immunomodulatory effects. The immune status of CRC patients varies significantly between early- and late-stage disease, with advanced-stage tumors often exhibiting greater immune suppression. However, due to inconsistent reporting in the included studies, it remains unclear whether CKI exerts different effects across disease stages. Stratified analyses should be conducted in future trials to determine whether CKI’s influence on immune function, treatment response, and chemotherapy toxicity differs between early- and advanced-stage CRC patients. Seventh, CKI’s potential therapeutic applications beyond CRC warrant further investigation. Although this meta-analysis focused on CRC, CKI’s immunomodulatory effects suggest possible benefits in other malignancies characterized by immune dysfunction. Future studies should explore CKI’s role in cancers such as hepatocellular carcinoma, lung cancer, and gastric cancer, potentially broadening its clinical utility.

## 5 Conclusion

Current evidence suggests that the combination of CKI with conventional chemotherapy may have beneficial effects on immune function, ORR, DCR, and chemotherapy-induced adverse reactions in CRC patients. However, given the variability in study quality and the lack of disease stage stratification, these findings should be interpreted with caution. Additionally, the short observation periods and absence of long-term follow-up data limit the understanding of CKI’s impact on survival and quality of life. High-quality, large-scale RCTs with extended follow-up are needed to further evaluate its long-term efficacy, safety, and broader clinical applicability in CRC management.

## Data Availability

The original contributions presented in the study are included in the article/Supplementary Material, further inquiries can be directed to the corresponding author.
